# Lack of Evidence for Neonatal Misoprostol Neurodevelopmental Toxicity in C57BL6/J Mice

**DOI:** 10.1371/journal.pone.0038911

**Published:** 2012-06-13

**Authors:** Claire M. Koenig, Cheryl K. Walker, Lihong Qi, Isaac N. Pessah, Robert F. Berman

**Affiliations:** 1 Center for Children's Environmental Health, University of California Davis, Davis, California, United States of America; 2 Department of Neurological Surgery, University of California Davis, Davis, California, United States of America; 3 Department of Obstetrics and Gynecology, University of California Davis, Davis, California, United States of America; 4 Division of Biostatistics, Department of Public Health, University of California Davis, Davis, California, United States of America; 5 Department of VM: Molecular Biosciences, University of California Davis, Davis, California, United States of America; Hôpital Robert Debré, France

## Abstract

Misoprostol is a synthetic analogue of prostaglandin E1 that is administered to women at high doses to induce uterine contractions for early pregnancy termination and at low doses to aid in cervical priming during labor. Because of the known teratogenic effects of misoprostol when given during gestation and its effects on axonal growth *in vitro*, we examined misoprostol for its potential as a neurodevelopmental toxicant when administered to neonatal C57BL6/J mice. Mice were injected subcutaneously (s.c.) with 0.4, 4 or 40 µg/kg misoprostol on postnatal day 7, the approximate developmental stage in mice of human birth, after which neonatal somatic growth, and sensory and motor system development were assessed. These doses were selected to span the range of human exposure used to induce labor. In addition, adult mice underwent a battery of behavioral tests relevant to neurodevelopmental disorders such as autism including tests for anxiety, stereotyped behaviors, social communication and interactions, and learning and memory. No significant effects of exposure were found for any measure of development or behavioral endpoints. In conclusion, the results of the present study in C57BL/6J mice do not provide support for neurodevelopmental toxicity after misoprostol administration approximating human doses and timed to coincide with the developmental stage of human birth.

## Introduction

The incidence of autism spectrum disorders (ASD) has increased over the last few decades [1]–[6]. In the majority of these cases the cause of ASD is unknown (idiopathic autism) but evidence is accumulating that environmental factors may contribute to the etiology of autism [4], [7]–[10]. Environmental factors under investigation include proximity to highways and associated air pollution [11], pesticide exposures [12], [13], maternal vitamin supplementation [14], [15], parental age [16]–[18], and maternal exposure to pharmacological agents during pregnancy and perinatal periods [19]–[21]. Pharmacological agents of interest include misoprostol [22]–[24], thalidomide [23], valproic acid (VPA) [25], [26], and tertbulaline [27], [28].

Misoprostol is a synthetic prostaglandin E1 analogue that was initially developed to treat peptic ulcers through its ability to decrease gastric acid secretion and increase mucosal protective properties [29], [30]. It was later determined that misoprostol was capable of producing cervical ripening and uterine contractions in pregnant women [31]–[33]. Because of these latter properties misoprostol is now commonly used in obstetric and gynecological practices to induce labor, or if administered at higher doses early in gestation to terminate pregnancy.

The use of misoprostol however is not without risk. There is evidence that prenatal exposure to high doses of misoprostol during the first or second trimester can lead to the occurrence of Möbius Syndrome, a disorder characterized by congenital palsy of the 6^th^ and 7^th^ cranial nerves [23]. Further in a study by Strömland et al. 7 out of 22 children with Möbius syndrome also displayed autistic characteristics [34], a significantly higher incidence then that seen in the general population [35]. This relationship between misoprostol exposure and the development of autistic features in Möbius Syndrome suggests the possibility that the use of misoprostol may be a developmental risk factor in autism [23], [36].

Dufour-Rainfray et al. have recently suggested that through different mechanisms several teratogens, including misoprostol, are able to modulate the expression of genes leading to developmental disorders with autistic like characteristics [36]. They theorize that altered gene expression can lead to deregulation of important neurodevelopmental processes, the same processes which have been shown to involve proteins encoded by genes mutated or altered in some patients with autism [37], [38]. Further recent findings by Tamiji et al. indicate that exposure to misoprostol can alter calcium homeostasis in nerve growth cones of mouse Neuro-2a cells, as well as cause retraction of developing neurites, providing a possible cellular mechanism by which misoprostol could influence development of the nervous system [39], [40]. Lastly, prostaglandins, including misoprostol, are known to regulate a wide variety of immunological processes, including cytokines IL-1, IL-4, IL-6, IL-8 and TNF α & β [41]–[44]. Because brain function and development are highly influenced by cytokine activity [45]–[47] and immune system abnormalities have been linked to autism [48]–[51], the possibility that misoprostol and other prostaglandins administered during labor may be a risk factor for ASD is a concern.

**Figure 1:**
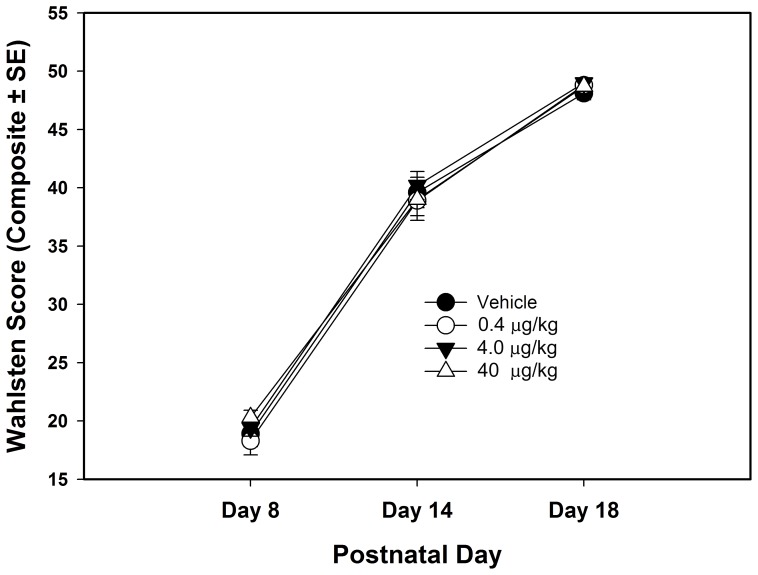
Neurodevelopmental composite score (sensory and motor development) for the four treatment groups. No group differences were statistically significant.

Preliminary findings from the CHARGE (*CHildhood Autism Risk from Genetics and the Environment*) Study, an ongoing case-control study of autism risk, reported that use of vaginal prostaglandins, including misoprostol, was more common among mothers of children with autism compared with controls, although this association failed to achieve statistical significance [52].

Therefore, we carried out the following study examining neonatal misoprostol exposure in mice for evidence of neurodevelopmental toxicity. In this study C57BL6/J mice were exposed to 0.4, 4, and 40 µg/kg misoprostol, s.c. on postnatal day (PND) 7. These doses were selected to span the range of clinical doses (∼0.33–35 µg/kg based on an average 75 kg body weight) used to induce labor [53], [54]. PND 7 was chosen because it approximates the developmental stage at human birth [55] when misoprostol is typically given to induce labor. A battery of behavioral tests was then administered to mice over the course of development to assess somatic growth as well as neurodevelopmental endpoints relevant to ASD, including social interactions, stereotypic behaviors, anxiety, and learning and memory [56]–[58].

## Materials and Methods

### Chemicals

Misoprostol (>98% purity) was purchased from Cayman Chemical Co. (Ann Arbor, MI). Following the laboratory procedures provided by the supplier, a solvent exchange from methyl acetate to dimethyl sulfoxide (DMSO. ≥99.9% purity) was conducted for preferred storage as recommended by Cayman Chemical Co. Dosing solutions were diluted from a stock solution with sterile physiological saline. Exposure to DMSO through administration of misoprostol did not exceed 0.722 mg/kg. This level of exposure is well below the levels used in studies which report neuroprotective properties of DMSO [59], [60].

**Figure 2:**
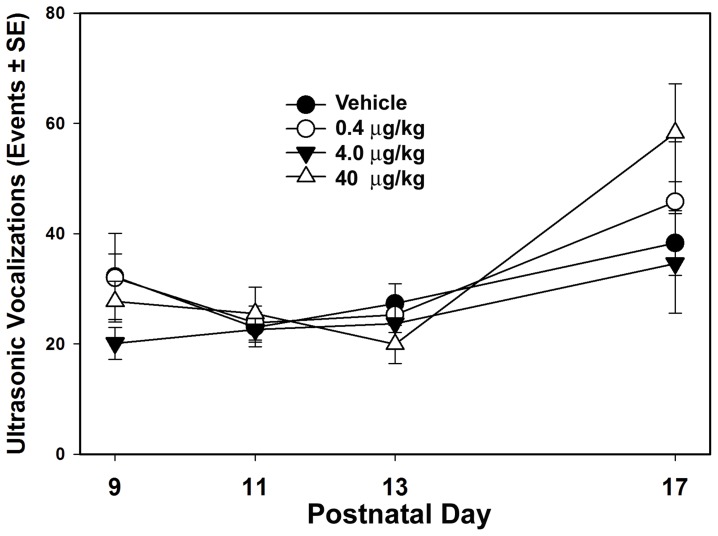
Number of bouts of ultrasonic vocalizations (USV) on postnatal days 9, 11 13 & 17. No group differences were statistically significant.

### Animals

Adult male and female C57BL6/J mice were purchased from Jackson Laboratory (Bar Harbor, ME) and maintained by the Center for Laboratory Animal Research (CLAS), at University of California, Davis. Mice were fed standard mouse chow (LabDiet, 5001 Rodent Diet) and were maintained on a 14 h light/10 h dark cycle, with the light cycle between 7AM to 9PM. Ambient temperature was maintained between 68±2°F and humidity between 40–60%.

After 1 week of acclimatization mice were mated and dams were checked daily for the presence of seminal plugs, after which the cages housing the pregnant dams were checked daily for birth of litters by viewing cage bottoms. On postnatal (PND) 7 pups in each litter were injected subcutaneously in the nape of the neck with either saline vehicle or 0.4, 4 or 40 µg/kg misoprostol. A within litter design was used, and when possible the full range of 4 doses for males and 4 doses for females was represented within a litter (i.e., 4 treatments by sex=8 injected mice). However, with litter sizes less than 8, pups were randomly assigned to one of the 4 treatments groups, with no single dose for males or females repeated within a litter. A total of 12 litters were used in this study. This allowed us to achieve final treatment group sizes of n=16 (8 male and 8 female mice per treatment group) with the same animals used for all behavioral testing. Mice were then marked for identification using non-toxic foot tattoos (Ketchum Manu. Inc., Brockville, ON, Canada).

All experimental procedures and protocols using mice were approved by the University of California, Davis Institutional Animal Care and Use Committee (IACUC) under protocol #16001.

### Behavioral Testing

Behavioral tests are described in the order that they were conducted, along with the approximate ages of the mice. All tests were conducted during the light phase of the light/dark cycle. Tests conducted prior to weaning on PND 21 were administered between 7am and 12pm. Tests conducted after weaning were administered between 12pm and 5pm.

#### 1. Growth and Reflex Assessments (PNDs 8, 14, 18)

Using a test battery described previously [61], somatic, sensory and motor development were assessed. Briefly, pups were removed from the home-cage and kept on a heating pad until testing. They were then individually observed using the following tests: righting reflex, cliff aversion, needle grasp, visual placing, vibrissa placing, ears open, ear twitch response, screen pull, screen cling/climb, narrow stick placing reflex, wide stick placing reflex, auditory startle, and popcorn behavior. Behaviors were scored as follows: 0 – no response; 1 – some response, weak and not coordinated; 2 – moderate or incomplete response; 3 – full and complete response. After completion of testing the pup was placed back in its home cage.

#### 2. Ultrasonic Vocalization (PNDs 9, 11, 13, 17)

Pup ultrasonic vocalization (USV) was measured by removing each pup from its litter and placing it in a plastic cup that was positioned under the microphone attached to the USV recording system (Ultravox, Noldus Instruments, City). Testing was carried out in a sound-attenuated testing chamber. Recording began within 15 seconds after the pup was removed from its mother. After the recording session the pup was kept separated from the rest of the litter until all pups were tested. The USV detector was set at 40 KHz and the audio filter was set at 8.5 and temperature inside the testing chamber was recorded at the end of each session. Recorded data were analyzed using the Noldus UltraVox program. Altered number and duration of USVs were used to asses pup distress.

#### 3. Weaning (PND 21)

On PND 21 pups were removed from dam and ear notched for identification and left with littermates until re-caging according to sex at puberty on PND 28.

#### 4. Sociability (PND 25–26)

Sociability and preference for social novelty were tested as described previously [62]. Testing was carried out in a 58×39 cm opaque Plexiglas chamber divided into three equal-sized compartments by two 22 cm high clear Plexiglas walls. Each wall had a door that allowed movement between the three compartments. During the an initial 10 minute habituation session the injected test mouse was placed in the central compartment and allowed to freely move between compartments. After habituation the test mouse was removed and a unfamiliar experimentally naïve male mouse was placed under a wire mesh cup (Galaxy Cup, Kitchen Plus, http://www.kitchen-plus.com) in one of the side compartments (side counterbalanced). The test mouse was then placed in the central compartment and allowed to freely move between compartments for another 10 minutes. Percent time spent in the side compartment with the unfamiliar mouse versus the opposite side chamber was calculated. A greater percentage of time spent in the chamber with the stranger mouse indicated a preference for social interaction, and was used as an index of “sociability” as previously described [62].

#### 5. Locomotor Activity (PND 27–28)

Locomotor activity was tested for 60 minutes using a TruScan Photo Beam open-field activity arena for mice (Coulbourn Instruments., Allentown, PA) as described previously [63]. The apparatus (27.5×27.5×37.5 cm) detects movements in the three geometric planes by recording infrared beam brakes resulting from mouse movement (e.g., horizontal movements, vertical movements, and distance traveled). Each animal was tested individually in the chamber. The data collected by the test session were then analyzed by TruScan software.

#### 6. Puberty Re-cage (PND 28)

All animals were re-caged according to their sex on postnatal day 28 with between 2–4 mice per cage.

#### 7. Elevated Plus Maze (PND 30)

Fear and anxiety were assessed using the elevated plus maze [64], [65]. The maze was made of opaque Plexiglas, with two open (30×5×0.25 cm) and two closed (30×5×6 cm) arms emanating from a central platform (5×5 cm), and elevated 60 cm above the floor. Each mouse was placed onto the central platform and allowed 5 minute to freely explore the maze. Distance traveled, number of entries into each arm, time in open vs. closed arm and latency to first arm entry were recorded by a video-tracking system (SMART, SD Instruments, San Diego, CA). Percent time spent on the open versus the closed arms of the maze was used as a measure of anxiety and fear.

#### 8. Pre-pulse inhibition (PND 32–33)

Pre-pulse inhibition (PPI) of the auditory startle response was measured to assess sensorimotor gating [66], [67]. Mice were placed individually into the auditory startle apparatus (SR-LAB, SD Instruments, San Diego, CA, USA) and allowed to acclimate to background white noise for 5 min. This was followed by a 20-minute PPI session consisting of 50 test trials, 10 each for five different trial types presented in a pseudorandom order with variable inter-trial intervals of 5–20 milliseconds (ms). Trial types included: 120 dB auditory stimulus alone, 120 dB stimulus with a 74, 82, or 90 dB pre-pulse auditory stimulus. All pre-pulses were 20 ms long and were presented 100 ms before the 120 dB stimulus. Broadband pink noise was used for the acoustic stimulus. The change in response from baseline to the 120 dB auditory stimulus was compared to the change in response from baseline to pre-pulsed 120 dB auditory stimulus to measure sensory motor gaiting.

#### 9. Spatial Memory and Learning in the Water Maze (PND 49–55)

Spatial memory and learning were tested in a water maze. The maze was 90 cm in diameter with a hidden 6 cm square escape platform submerged 1 cm below the surface of the water. Water temperature was maintained at 21°C. Maze performance was monitored using an automated tracking system (Polytrack, San Diego Instruments, Ca, USA). Before training began, mice were given a single trial in which they were allowed to swim to a visible escape platform raised 2 cm above the water surface. This was immediately followed by four 90 sec training trials with the platform hidden 1 cm below the surface of the water. Briefly, mice were placed in the maze at one of three quadrants not containing the platform, and were allowed to swim in order to locate and mount the escape platform. These four test trials were then repeated over four consecutive days. Latency to mount the platform, swim distance, swim speed, and time spent floating were measured. Animals who failed to reach the platform during any trial received the maximum 90 s score for latency. Inter-trial intervals were 10 minutes during which the mouse waited in a warming cage. On the fifth day of training a “probe trial” was given in which the platform was removed. Mice were released at the center of the quadrant opposite the quadrant where the platform was previously located, and the animals were allowed to swim freely for 90 seconds. Percent time spent swimming in the former escape platform quadrant was used to assess memory for spatial location of the platform.

### Histological Analysis

Once behavioral testing was complete a subset of mice were sacrificed by an intraperitoneal injection of Euthasol (100 mg/kg) for unbiased stereological examination to determine whether misoprostol injections resulted in a possible loss of CA1 hippocampal neurons. A total of 23 males and 10 females were used for these analyses. There were n=6, 5, 6, and 6 male mice in the vehicle, 0.4, 4 and 40 µg/kg groups, respectively. For the female mice there were n=5 in the vehicle and n=5 in the high dose 40 µg/kg group. The hippocampus was selected for study because it sensitive to a variety of developmental neurotoxins [68]. Mice were perfused with 20 ml of 0.1 M phosphate buffer (PB) (pH 7.4) followed by a 20 minute gravity fed perfusion with 4% paraformaldehyde (PFA) in 0.1 M sodium phosphate buffer. After perfusion brains were removed and post-fixed for 1 hour in 4% PFA, followed by cryoprotection in 10% sucrose in 0.1 M PB solution for 1 hour and a 30% sucrose 0.1 M PB solution for 24 hours. After cryoprotection brains were flash frozen in dry ice and stored at −80°C until sectioning. Brains were blocked and sectioned at 50 µm on a sliding microtome (AO model 860). Slices were preserved in 0.1% sodium azide in 0.1 M PB until mounting. Every 5^th^ section was mounted starting approximately at Bregma −1.46 and ending at approximately Bregma −2.92 based on the stereotaxic atlas The Mouse Brain in Stereotaxic Coordinates, Second Edition [69], and cresyl violet stained. A total of 7 mounted sections for each subject were used for unbiased stereological analysis of neuron number in the CA1 subregion of the hippocampus using the optical fractionator probe (StereoInvestigator, Microbrightfield, Williston, VT).

### Statistical Analysis

Data in figures represent mean ± standard error (SE) of the mean. Statistical analyses were carried out using version 18 of SPSS (SPSS, Chicago, IL) and version of 9.2 of the SAS programming language (SAS Institute, Cary, NC). For statistical analyses 8 male pups and 8 female pups from each treatment group were analyzed with total N=16 for each treatment group. Data were analyzed using a mixed effects model that included treatment and sex, with litter as a random effect. Repeated measurements over time for each pup were considered by using the autoregressive-1 (AR(1)) covariance structure. Individual *post hoc* group comparisons were made using the Tukey-Kramer test for multiple comparisons. Data were examined for homogeneity of variance, and when assumptions of homogeneity of variance were not met, data were analyzed using the Kruskal-Wallace nonparametric analysis, followed by individual *post hoc* group comparisons using the Mann-Whitney U adjusted for multiple comparisons. The minimum level set for statistical significance was P<0.05.

## Results

Treatment groups did not differ significantly in body weight on the day of drug treatment (i.e., PND 7), or across the period of behavioral testing. No signs of overt toxicity (e.g., lethargy, vocalizations) were observed across treatment groups.

### Behavioral Tests

**Figure 3:**
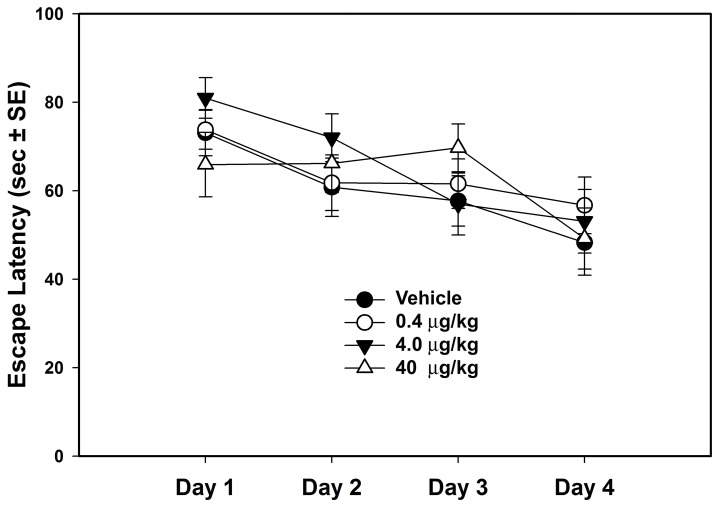
Mean escape latency in the Morris water maze for the four treatment groups. No group differences were statistically significant.

A total sensory and motor developmental score was calculated from the individual tests and the results are shown in Figure 1. Statistical analysis of the Wahlsten neurodevelopmental test battery did not show significant differences across treatment groups in sensory or motor development (F_3,64_=.317, P=.813, Effect Size=.009). Figure 2 shows the number of bouts of USV for each group across PNDs 9, 11, 13 & 17. There were no statistically significant effects of misoprostol treatment on USVs (F_3,64_=1.020, P=.390, Effect Size=.049). Average escape latencies during training in the Morris water maze are shown in Figure 3, but there were no significant differences between groups (F_3,64_=.267, P=.849, Effect Size=.013). Table 1 shows the mean +/− SEs, F values, probabilities and effect sizes for the main effect for the remaining behavioral tests. As shown in Table 1, there were no statistically significant differences in these tests between treatment groups.

**Table 1:** Summary of main effects for statistical analyses of behavioral performance across treatment groups.

Test	Parameter	Test Day	Dose	N	Mean ± SE	F3,63^2^, P^3^ & ES^4^ Values
Sociability	% Time Socializing	PND 25^1^	0	16	58.5±4.0	F=.192
			0.4	16	54.1±3.4	P=.901
			4	16	56.6±4.5	ES=.010
			40	16	55.7±4.9	
						
Locomotor	Total Distance Traveled	PND 27	0	16	5478±404	F=.633
			0.4	16	5047±280	P=.597
			4	16	4907±301	ES=.031
			40	16	5382±360	
						
EPM	% Time in Open Arm	PND 30	0	16	21.1±2.9	F=.997
			0.4	16	16.5±1.2	P=.401
			4	16	20.5±1.8	ES=.047
			40	16	20.5±2.2	
						
PPI	% Change in Startle from 90 db	PND 32	0	16	16.3±5.6	F=.948
			0.4	16	23.3±3.9	P=.423
			4	16	25.1±2.4	ES=.045
			40	16	20.9±3.1	

1 PND – postnatal day.

2 F – F ratio from ANOVA.

3 P – probability.

4 ES – eta squared effects size estimate.

### Hippocampal Cytoarchitecture

**Figure 4:**
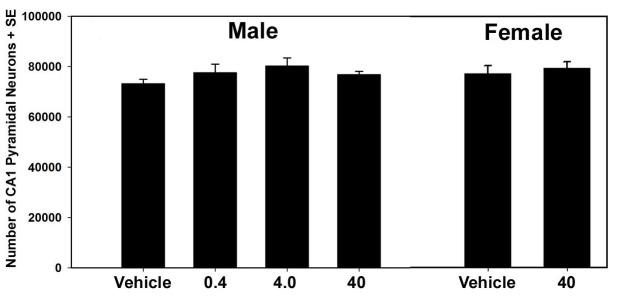
Number of hippocampal CA1 pyramidal neurons across treatment groups and sex. Four treatment groups (i.e. vehicle, 0.4, 4.0 & 40 µg/kg misoprostol, s.c.) were analyzed for male mice, and two treatment groups, the vehicle and high 40 µg/kg dose group, were analyzed for females. No significant treatment effects, sex differences or sex by treatment interaction were found.

No gross morphological abnormalities in structure of the CA1 region of the hippocampus of male or female mice exposed to misoprostol were found from examination of cresyl violet stained brain sections. As shown in Figure 4, stereological analysis of the number of pyramidal neurons in the CA1 subregion of the hippocampus did not reveal any statistically significant differences among treatment groups for males (F_3,19_=1.44, p=0.26) or females (F_1,8_=0.27, p=0.62). There was no significant difference between males and females (F_1,27_=1.54, p=0.26) and the sex by treatment interaction was also not significant (F_1,27_=0.07, p=0.79).

## Discussion

The results of the present study failed to reveal significant neurodevelopmental effects of neonatal subcutaneous injection with 0.4, 4 or 40 µg/kg misoprostol on PND 7 in C57BL/6J mice. This was true for tests of pre-weaning sensory and motor development and adult locomotor activity. Tests of social interaction and anxiety also failed to indicate any significant neurodevelopmental effects of misoprostol. There was no evidence for deficits in spatial learning or memory in the Morris water maze. Finally, no gross effects on brain morphology were observed, and there was no loss of hippocampal neurons in mice exposed to misoprostol. The lack of neuronal loss in the hippocampus is consistent with reports that misoprostol can be neuroprotective and reduce apoptosis in several different cell types [70]–[73]. However, the possibility remains that the fine structure of the nervous system (e.g., dendritic spines, dendrite branching) could be affected by misoprostol, and future research on this possibility would be interesting in light of studies showing that misoprostol can induce neurite retraction *in vitro* [39], [40].

While it is possible that statistical power was insufficient to detect significant group differences, group sizes fell within the range that have been recommended for behavioral experiments in mice [57]. In addition, effect sizes calculated for each of the tests were small and did not support rejecting the null hypothesis of no differences among treatment groups. Because our behavioral test battery was not exhaustive there is the possibility that under different testing conditions treatment effects may have been observed. However, the test battery used in the current study was chosen, in part, on the recommendations by Moy et al. concerning appropriate behavioral tests for animal models of autism [56].

In the present study, the choice of PND 7 was to approximate the time of human infant exposure to misoprostol which typically occurs at the time of birth [55]. However, treatment with misoprostol on PND 7 may have been too late in development to affect critical windows of neurodevelopmental vulnerability [23], [36]. Studies evaluating developmental disorders linked to maternal inflammation indicate that the most sensitive time period of altered neurodevelopment occurs during gestation [74]. Further there is in vitro evidence that misoprostol specifically disrupts calcium homeostasis during early neuronal development resulting in growth cone retraction [39]. Therefore future research to evaluate earlier neonatal time points of exposure should be conducted to determine whether the immature brain is more vulnerable to the effects of misoprostol.

Another concern with the current study is whether the subcutaneous route of drug administration resulted in sufficient levels of misoprostol in the mouse pup (e.g. brain) to alter behavioral development. The three log doses examined in this study were meant to cover the range of exposures experience by woman given misoprostol to induce labor with the highest dose, 40 µg/kg, exceeding the level used in pre-term abortion. Because exposure to misoprostol occurs indirectly in the human infant as a result of administration to the mother at the time of birth, it could be assumed that direct exposure of pups to misoprostol by s.c. injection would result in a higher concentration of misoprostol within the pup, including brain, when compared to the unborn child.

The lack of evidence in the current study for neurobehavioral toxicity after in vivo exposure to misoprostol differs from the results of in vitro studies by Tamiji et al. [39], [40]. This could be due to several factors such as differences in exposure levels and physiological differences between in vivo and in vitro models. In the study by Tamiji et al. [39] Neuro-2a cells directly exposed to 0.1 and 1 µM of misoprostol while in the current study neonatal mice were exposed to 0.4, 4 and 40 µg/kg s.c.; thus it would be expected that the actual exposure of neurons to misoprostol would differ between the in vitro and in vivo models. Further the Neuro-2a cell line used in the Tamiji et al. studies [39], [40] has been shown to differ importantly from primary cultures of intact neurons [75]. Specifically Neuro-2a cells showed a 20% decrease in expression of voltage-gated sodium channels (VGSC) and an absence of N-methyl-D-aspartate receptors (NMDAR) compared to intact neurons, which led to differences in sensitivity to chemicals known to produce neurotoxicity. This underscores the need to exercise caution in interpreting data from in vitro studies, including neuroblastoma cell lines. In addition, Taniguchi et al. demonstrated that misoprostol was neuroprotective, and not neurotoxic, at 50 and 500 µg/kg when given to PND 7 old rat pups in an in vivo model of Hypoxic-Ischemic Encephalopathy (HIE) [76].

In summary, the present results do not provide support for neurodevelopmental toxicity by misoprostol in neonatal mice at the range of doses examined and when given at a time approximating human birth. However, they do not prove that misoprostol plays no role in the etiology of neurodevelopmental disorders, including ASD. In fact, the timing of misoprostol exposure during gestation appears to be critical [23], [36]. When high-dose misoprostol is given unsuccessfully early in gestation to induce abortion, rather at the time of birth to induce labor, the surviving fetus may develop Möbius syndrome, a disorder characterized by congenital facial nerve paresis, congenital limitation of abduction and a co-occurrence of autism like symptoms [22], [23], [77]. However, there is no convincing evidence that when given at the time of birth misoprostol is a risk factor for neurodevelopmental disorders, including autism. This is supported by both the current results and the results of the CHARGE study in humans. Although the results of the current study are negative concerning developmental neurotoxicity of misoprostol, they are important none-the-less because of the large number of environmental and teratogenic agents currently under suspicion for their role in developmental disorders, and the need for scientific data that can help focus future research efforts.
